# Grape Seed Procyanidin Reversal of P-glycoprotein Associated Multi-Drug Resistance via Down-regulation of NF-κB and MAPK/ERK Mediated YB-1 Activity in A2780/T Cells

**DOI:** 10.1371/journal.pone.0071071

**Published:** 2013-08-15

**Authors:** Bo-xin Zhao, Ya-bin Sun, Sheng-qi Wang, Lian Duan, Qi-lu Huo, Fei Ren, Guo-feng Li

**Affiliations:** 1 Department of Pharmacy, Nanfang Hospital, Southern Medical University, Guangzhou, Guangdong, China; 2 GCP Office, Nanfang Hospital, Southern Medical University, Guangzhou, Guangdong, China; Northwestern University Feinberg School of Medicine, United States of America

## Abstract

The expression and function of P-glycoprotein (P-gp) is associated with the phenotype of multi-drug resistance (MDR), leading chemotherapy failure of patients suffered with cancer. Grape seed procyanidin(GSP) is a natural polyphenol supplement with anti-inflammatory effect. Present study assessed a new use of GSP on the MDR reversal activity and its possible molecular mechanisms in MDR1-overpressing paclitaxel resistant ovarian cancer cells. Our results showed GSP significantly enhanced the cytotoxicity of paclitaxel and adriamycin in paclitaxel resistant A2780/T cells but its parental A2780 cells. Furthermore, GSP strongly inhibited P-gp expression by blocking MDR1 gene transcription, as well as, increased the intracellular accumulation of the P-gp substrate rhodamine-123 in A2780/T cells. Nuclear factor-κB(NF-κB) activity, IκB degradation level and NF-κB/p65 nuclear translocation induced by lipopolysaccharide (LPS) and receptor activator for nuclear factor-κB ligand (RANKL) were markedly inhibited by pre-treatment with GSP. Meanwhile, GSP inhibited MAPK/ERK pathway by decreasing the phosphorylation of ERK1/2, resulting in reduced the Y-box binding protein 1 (YB-1) activation with blocking its nuclear translocation. Moreover, the up-regulation of P-gp expression, the activation of AKT/NF-κB and MAPK/ERK pathway induced by LPS was attenuated by GSP administration. Compared with PDTC and U1026, inhibitor of NF-κB and MAPK/ERK respectively, GSP showed the same tendency of down-regulating NF-κB and MAPK/ERK mediated YB-1 activities. Thus, GSP reverses P-gp associated MDR by inhibiting the function and expression of P-gp through down-regulation of NF-κB activity and MAPK/ERK pathway mediated YB-1 nuclear translocation, offering insight into the mechanism of reversing MDR by natural polyphenol supplement compounds. GSP could be a new potential MDR reversal agent used for combination therapy with chemotherapeutics in clinic.

## Introduction

Plenty of cancer cells develop resistance against their chemotherapeutic agents which are structurally and mechanistically various. For example, paclitaxel and adriamycin, are widely used in ovarian cancer chemotherapy treatment, come out unsatisfactory only because the tumor lost the sensibility to the chemotherapeutic agents [1], which currently known as multi-drug resistance (MDR). Intrinsic and acquired MDR mainly results from the overexpression of cell membrane-bound ATP-binding cassette (ABC) transporters, which actively extrude a variety of chemotherapeutic drugs out of the cancer cells [2]. Importantly, P-glycoprotein (p-gp), encoded by MDR1 gene, is able to transport a wide range of anticancer agents such as the anthracyclines, vinca alkaloids, taxanes, and epipodophyllotoxins [3], thereby may be responsible for intrinsic and acquired drug resistance in numerous human cancers. Recently, P-gp associated MDR is proved to be effectively overcome by either blocking its drug-pump function or inhibiting its expression [4]. Thus, suppression of P-gp function and expression may certain reverse the P-gp associated MDR in cancer cells that comes to increase the efficacy of chemotherapy.

Since P-gp associated MDR was first identified exceed semi-century ago, researches on new effective P-gp inhibitors have attracted extensive attention worldwide. The discovery of verapamil reversal MDR by blocking P-gp in 1980 s led to a wave of P-gp inhibitor development, various agents including designed compounds have been reported to suppress P-gp [5], [6]. However, most of these agents necessitated high doses which caused serious side-effects and the intrinsic cytotoxicity, especially the designed compounds, by dose-limiting toxicity, consequently, relevant clinical trial results disappointingly. Although new generation of P-gp inhibitors have been developing, novel approaches in overcoming P-gp/MDR1 mediated MDR by blocking its function and expression are still needed. In this case, natural supplement agents are gaining increasing interest in cancer supplementary therapy.

MDR1 expression has been studied in a certain cancer cells, including human ovarian cancer cells A2780 and its multidrug resistant subline A2780/T [7], [8], [9]. Molecularly, the P-gp/MDR1 expression is mediated by nuclear factor κ-light-chain-enhancer of activated B cells (NF-κB) [10], [11], [12], cylooxygenases-2 [13], CYP3A4 [14], the mitogen-activated protein kinase (MAPK) pathway [15], [16], [17], and phosphoinositide 3-kinase (PI3K) [10], [18]. Among these, NF-κB and MAPK/ERK pathway are the most important factors in terms of their molecular mechanisms for inducing MDR. The NF-κB pathway responds actively to MDR1 induction due to its activation by the generation of reactive oxygen species, the activation of IκB kinase, and the degradation of IκB [19]. Besides, NF-κB is bound at nucleotide position −159 of the MDR1 promoter mediating the transcription of MDR1 [20]. Similarly, the MAPK pathway, comprising extracellular signal-regulated kinase (ERK), c-Jun NH2-terminal kinase (JNK)/stress-activate protein kinase (SAPK), and p38MAPK subfamilies, also plays critical roles in the transmission of signals provided by various kinds of stimulus to regulate the expression of MDR1. Recently, a number of studies showed that the over-expression of P-gp appears to be closely associated with the nuclear localization of Y-box binding protein 1(YB-1) in various solid tumors such as breast cancer, ovarian cancer, prostate cancer, and osteosarcoma [21,22,23]. Meanwhile, Coles et al demonstrated that MAPK/ERK pathway regulated the phosphorylation of YB-1 by ERK phosphorylation [24]. However, the interaction of natural flavonoids agents and MAPK/ERK mediated YB-1 activity has not been reported yet.

**Figure 1:**
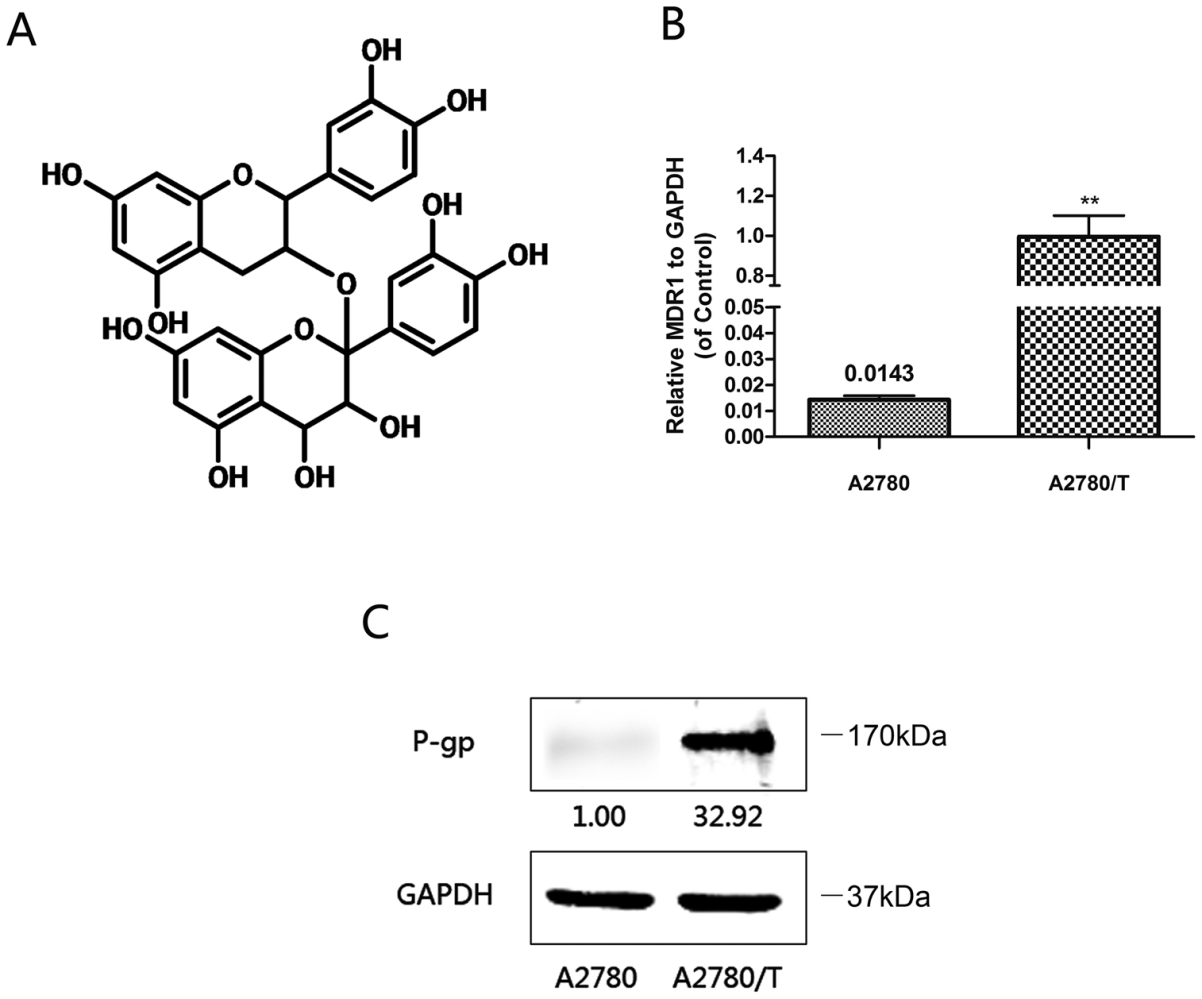
The structure of GSP and the characterization of A2780 and A2780/T cells. (A) The structure diagram of catechin-3-O-2-leucocyanidin (GSP). (B) The expression of MDR1 mRNA in A2780 and A2780/T cells was analyzed by RT-qPCR. MDR1 gene was overexpressed in the resistant cells (A2780/T). **Significantly different from A2780 cells (P<0.01). (C) The protein product of MDR1 gene in A2780 and A2780/T cells was analyzed by Western blotting. P-gp protein was overexpressed in A2780/T cells.

Grape seed procyanidin (GSP), a class of polyphenol flavonoids compounds, naturally contained in fruits, vegetables, nuts, seeds, flowers and barks, is reported to exhibit a wide range of biological effects including antibacterial, antiviral, anti-inflammatory, antiallergic and vasodilatory actions [25], [26], [27] (Fig. 1A). GSP as a main ingredient of Pyconogenol (PYC, Horphag Research Ltd, UK) is used as a popular dietary supplement to overcome inflammatory and degenerative diseases and wound healing [28], [29]. Recent years, GSP is most widely studied in terms of their bioactivities. It's found that GSP could inhibit the function of P-gp on blood-brain barrier [30]. But the role of GSP in reversing cancer cells MDR by inhibiting P-gp expression as well as the mechanism remains unreported. Therefore, we investigated whether GSP can enhance the cytotoxicity of paclitaxel along with adriamycin and down-regulate possibly the expression with the transcription of P-gp/MDR1 in A2780/T cells. Furthermore, we demonstrated whether the MDR1 expression inhibited by GSP has involved inhibitory effect of NF-κB activity and MAPK/ERK pathway mediated YB-1 in A2780/T cells.

## Materials and Methods

### Reagents

GSP(>98%) was obtained from Aladdin Reagent Co.(Shanghai, China), of which the typical molecular structure was shown in Fig. 1A. Anti-P-gp monoclonal antibody and anti-NF-κB (p65) antibody were purchased from Santa Cruz Biotechnology, Inc. (California, USA). Anti-phosphorylated IκBα, anti-IκBα and anti-YB-1 were obtained from Epitomics, Inc. (California, USA). PDTC was obtained from Beyotime Institute of Biotechnology, Inc. (Shanghai, China). U1026 was provided from Cell Signaling Technology, Inc. (Danvers, MA). Antibodies against GAPDH, phosphorylated ERK1/2, total ERK1/2, phosphorylated AKT, total AKT were purchased from Bioworld Technology, Inc. (Minnesota, USA). Receptor activator for nuclear factor-κB ligand (RANKL) was obtained from ProSpec-Tany TechnoGene, Co.(Israel). Paclitaxel was purchased from Tianfeng Technology, co.ltd. (Xi'an, China). Adriamycin was purchased from Meilun Biology technology, co. ltd. (Dalian, China) Rhodamine 123 (Rho-123), 1-(4,5-dimethylthiazol-2-yl)-3,5-diphenyltetra-zoliumbromide (MTT), verapamil, lipopolysaccharide (LPS) and other chemicals were obtained from Sigma Chemical Co.(St. Louis, MO).

### Cell culture

A2780 human ovarian cancer cells and its multidrug resistant subline A2780/T were obtained by KeyGEN Biotech Co. (Nanjing, Jiangsu, China), where they were characterized by mycoplasma detection, cell vitality detection and authenticated by morphologic inspection. All cells were immediately expanded and frozen into multiple aliquots, such that they could be restarted of the early-passage stocks. The A2780 cells and A2780/T cells were grown in Dulbecco's modified Eagles's medium and Roswell Oark Memorial Institute 1640 respectively with 10% foetal bovine serum with 100 U/ml of penicillin, and 100 μg/ml of streptomycin. Additionally, the medium of A2780/T cells contained 800 ng/ml of Paclitaxel for maintaining the activity of MDR. Both cell lines were cultured at 37°C in a humidified CO_2_ incubator. The cells were cultured for 1 week in GSP-free medium prior to use in experiments.

### Measurement of cell viability

The cells were plated in 96-well plates, and cell viability was determined by the conventional MTT reduction assay. After 24 h incubation, various paclitaxel and adriamycin concentrations were added to the wells with or without different concentrations of GSP, 40 μM Verapamil was used as a positive control, and the plates were incubated for 48 h. In order not to exceed the absorbance linearity range, a suboptimal wavelength (490 nm) was used to read the samples. The IC_50_ was calculated from survival curves using the Bliss method [31]. The degree of resistance was estimated by dividing the IC_50_ for the MDR cells by that of the parental sensitive cells; and the fold-reversal factor of MDR was calculated by dividing the IC_50_ of the anticancer drug in the absence of GSP by that obtained in the presence of GSP.

### Rhodamine-123 accumulation assay

A2780 and A2780/T cells were seeded onto 48-well plates at a density of 5×10^4^ cells per well. The cells were pretreated with various concentrations of GSP or 40 μM verapamil for 24 h. Verapamil was used as a positive control for MDR inhibition [32]. After pretreatment, cells were incubated with 10 μM Rhodamine-123 (Rho-123) in culture medium in the dark at 37°C in 5% CO_2_ for 90 min. After incubation, the medium was removed, and all monolayers were washed three times with ice-cold PBS and then dissolved in 1% Triton X-100. The fluorescence of Rho-123 was determined using a fluorescence spectrophotometer (SpectraMax M5, MD, CA, USA) with an excitation wavelength of 488 nm and emission wavelength of 530 nm. The concentration of Rho-123 in each sample was determined from the fluorescence measurements by the construction of Rho-123 standard curve. The concentration of intracellular Rho-123 was normalized by the amount of protein and expressed as nmol/g protein as described previously [33].

### RNA isolation and RT-PCR analysis

RT-PCR was performed to evaluate MDR1 mRNA expression after GSP treatment. Total RNA was isolated with an RNA isolation plus kit (Takara Shuzo, Kyoto, Japan), according to the manufacturer's protocol, and the RNA quality was confirmed by an optical density measurement of A260/A280>1.8. PCR product formation was monitored continuously using ABI 7500 software, version 2.0.4 (Applied Biosystems, Foster City, CA, USA). The cDNA was amplified by 40 PCR cycles of 95°C for 5 s and 60°C for 34 s, using SYBR® Premix Ex Taq^TM^ (Takara Shuzo, Kyoto, Japan) and the following primers: 5′-TGGGGCTGGACTTCCTCTCATGATGC-3′ and 5′-GCAGCAACCAGCACC CCAGCACCAAT-3′ for MDR1, and 5′-AGAAGGCTGGGGCTCATTTG-3′ and 5′-AGGGGCCATCCACAGTCTTC-3′ for GAPDH. The PCR products were electrophoresed in 1.5% agarose gels, visualized by ethidium bromide staining, and photographed under ultraviolet light. The quantity of each transcript was calculated as described in the value of Ct and normalized to the value of GAPDH.

### Western blot analysis

After treatment, the cells were collected and washed with phosphate-buffered saline (PBS). The harvested cells were lysed on ice for 30 min in 100 μL of lysis buffer and centrifuged at 15 000×g for 30 min. The supernatants were collected, and protein concentrations were determined using a BCA protein assay kit (Pierce, Rockford, IL, USA). Aliquots of the lysates (30 mg of protein) were boiled for 5 min and electrophoresed in 10% SDS-polyacrylamide gels. The separated proteins were transferred onto nitrocellulose membranes, which were incubated with antibodies against P-gp/MDR1, phospho-ERK1/2, ERK1/2, phospho-Akt, Akt, phospho-IκBα, IκBα, and GAPDH, followed by incubation with secondary anti-mouse or anti-rabbit antibodies.

### Preparation of nuclear and cytosolic extracts

Nuclear extracts were prepared with a commercial kit according to the manufacturer's instructions (KeyGEN, Nanjing, China). All steps were performed on ice or at 4°C, unless stated otherwise. Protease inhibitors (10 mg·mL^−1^ aprotinin and 10 mg·mL^−1^ leupeptin) and reducing agents (1 mM dithiothreitol and 1 mM phenylmethylsulphonyl ﬂuoride) were added to each buffer just prior to use. Brieﬂy, cells were incubated in 10 volumes of hypotonic Buffer A (20 mM HEPES, pH 7.9, 1.5 mM MgCl_2_, 10 mM KCl) on ice for 15 min and homogenized. Nuclei were recovered by centrifugation at 16000×g for 5 min, and the supernatant was collected as the cytosolic extracts. The nuclei were extracted using Buffer C (20 mM HEPES, pH 7.9, 25% glycerol, 420 mM NaCl, 0.2 mM EDTA, 1.5 mM MgCl_2_) for 40 min on ice. Insoluble material was removed by centrifugation at 16 000×g for 10 min, and the supernatant was used as the nuclear extract.

### Immunofluorescence

Cells were plated onto 24-well plate with slides and incubated A2780/T cells overnight. The cells were pro-incubated with 40 μM GSP or drug-free medium for 24 h, and then exposed to the serum-free medium absence or presence 1 μg/ml LPS or 50 ng/ml RANKL for 30 min. All washes were done with PBS and all incubations were completed at room temperature unless otherwise specified. The cells were washed three times, and fixed with pre-frozen menthol for 30 min at −20°C. Then permabilised with 0.25% Triton X-100 for 15 min and washed three times. Cells were stained with incubated with NF-κB(p65) antibody overnight at 4°C after blocking cells by 1% BSA for 1 h at 37°C. Then washed for another three times followed by incubating with Cylight 488 AffiniPure Goat Anti-Rabbit IgG(H+L) for 1 h. Finally, they were cover-slipped using mounting media containing DAPI and stored at 4°C before microscopy.

### Transient transfection and luciferase assay

The promoter activity of the cells was determined using Luciferase Reporter Assay System (Pierece). First, the cells were plated in 24-well plates at a density of 1×10^5^ cells per well and transiently co-transfected overnight with the hMDR1-Luc and pCMV-Gussia (Gussia luciferase expression for normalization) (Pierece) using TurboFect^TM^ Transfection Reagent (Fermentas), then exposed to GSP with or without 10 μM paclitaxel for 24 h. Finally, luciferase activities in cell mediums were measured using a luminometer (SpectraMax M5, MD, CA, USA). The MDR1 promoter-driven Cypridina luciferase activities were normalized to Gussia luciferase activity.

### Statistical analysis

All experiments were repeated at least three times. For quantitative analysis, the sum of the density of bands corresponding to protein blotting with the antibody under study was calculated, and was normalized based on the amount of house-keeping protein. The differences between treatment groups were determined by One-way ANOVA and Student-Newman-Keuls(SNK) test was used for comparison among groups analysis. Statistical significance was accepted for P-values<0.05.

## Results

### Characterization of A2780 and A2780/T cells

To confirm the reported overexpression of MDR1 in A2780/T cells, the MDR1 mRNA and protein expression levels were compared between A2780/T and A2780 cells by RT-PCR and Western blot analysis respectively, which showed that MDR1 mRNA and protein levels were both overexpressed in A2780/T cells(Fig. 1B and C). A2780 and A2780/T cells were exposed to paclitaxel(0∼100 μM) and adriamycin (0∼80 μM) for 48 h. The IC_50_ value in A2780/T and A2780 cells was significantly (P<0.001) difference of paclitaxel (100.36±1.68 μM vs. 0.23±0.02 μM) and adriamycin (15.08±0.39 μM vs. 0.65±0.02 μM), indicating that A2780/T cells were the paclitaxel resistant subline of A2780, and MDR1 was overexpressed in A2780/T cells.

### GSP increased paclitaxel and adriamycin toxicity in A2780/T cells

**Figure 2:**
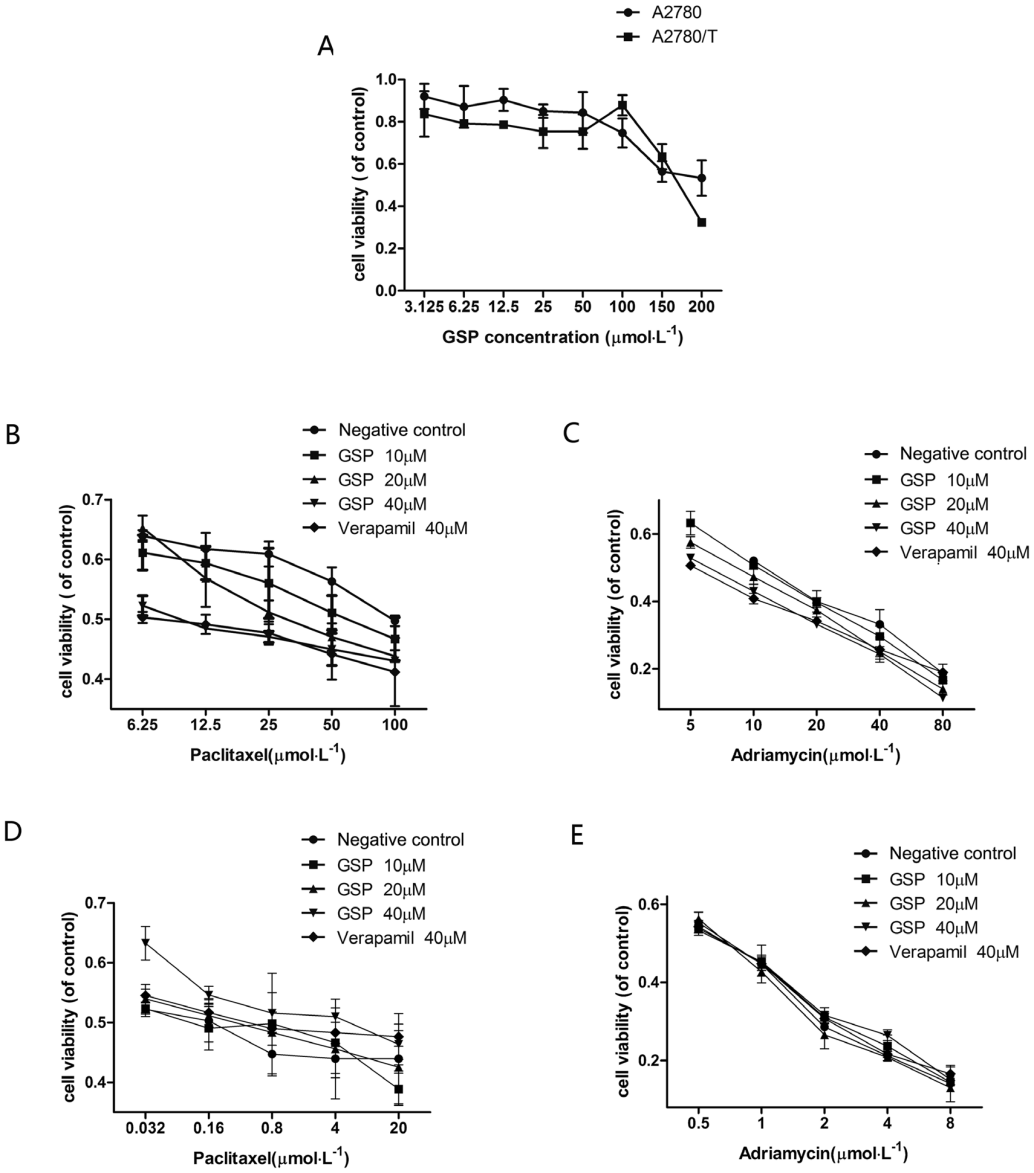
GSP increased the cytotoxicity of paclitael and adrimycin in A2780 and A2780/T cells. (A) The cytotoxicity of GSP (3.125∼200μM) in A2780 and A2780/T cells was determined by MTT assay. Each point shows the mean ± SD of three independent experiments, performed in triplicate. Effects of GSP and paclitaxel (B and D) and adriamycin (C and E) in A2780/T (B and C) and A2780 (D and E). Cells were pretreated with or without 10 to 40 μM GSP followed by incubation with various concentrations of paclitaxel for an additional 48 h. The viability of cells was determined by MTT assay. Each point shows the mean ± SD of thee independent experiments, performed in triplicate.

The viability of A2780 and A2780/T cells against GSP (3.125∼200 μM) treated for 48 h was determined using the MTT assay, which suggested that the concentration groups of GSP used in the study (0∼40 μM) had insignificant effect to the cells viability (Fig. 2A). Then the effect of GSP on paclitaxel and adriamycin cytotoxicity was examined in A2780 and A2780/T cells. The cells were treated in GSP (0∼40 μM) combining with various concentrations of paclitaxel (0∼20 μM for A2780, and 0∼100 μM for A2780/T) and adriamycin (0∼8 μM for A2780, and 0∼80 μM for A2780/T) for 48 h. The viability of the cells were analyzed by the MTT assay. As shown in Table 1 and Figure 2, GSP significantly enhanced the cytotoxicity of paclitaxel (Fig. 2B) and adriamycin (Fig. 2C) against A2780/T cells, but had almost no effect on the cytotoxicity of paclitaxel (Fig. 2D) and adriamycin (Fig. 2E) against A2780 cells. These results indicate that GSP selectively increased the cytotoxic effect of paclitaxel and adriamycin in A2780/T cells in a dose-dependent manner.

**Table 1:** Effect of GSP on reversing mult-drug resistance (IC_50_± SD μM (fold-reversal), n = 3).

Compounds	A2780	A2780/T
Paclitaxel	0.23±0.02(1.00)	100.36±1.68(1.00)
Paclitaxel+10μM GSP	0.22±0.02(1.05)	53.63±3.93(1.87)**
Paclitaxel+20μM GSP	0.24±0.01(0.96)	41.12±6.63(2.44)**
Paclitaxel+40μM GSP	0.23±0.01(1.00)	11.36±1.13(8.83)**
Paclitaxel+40μM Verapamil	0.25±0.06(0.92)	11.48±1.44(8.74)**
Adriamycin	0.65±0.02(1.00)	5.08±0.39 (1.00)
Adriamycin+10μM GSP	0.66±0.02(0.98)	11.43±0.96(1.32)**
Adriamycin+20μM GSP	0.66±0.03(0.98)	8.56±0.73(1.76)**
Adriamycin+40μM GSP	0.66±0.02(0.98)	6.30±0.38(2.39)**
Adriamycin+40μM Verapamil	0.65±0.02(1.00)	4.99±0.18(3.02)**

The viability of cells was determined by MTT assay as described in 2.2. Data are the means ± standard deviations (SD) of at least three independent experiments performed in triplicate. The fold reversal of MDR was calculated by dividing the IC_50_ for cells with the paclitaxel and adriamycin in the absence of GSP or verapamil by that obtained in the presence of inhibitor. ** P<0.01 versus that obtained in the absence of GSP or verapamil.

### GSP down-regulated MDR1 mRNA and protein expression in A2780/T cells

**Figure 3:**
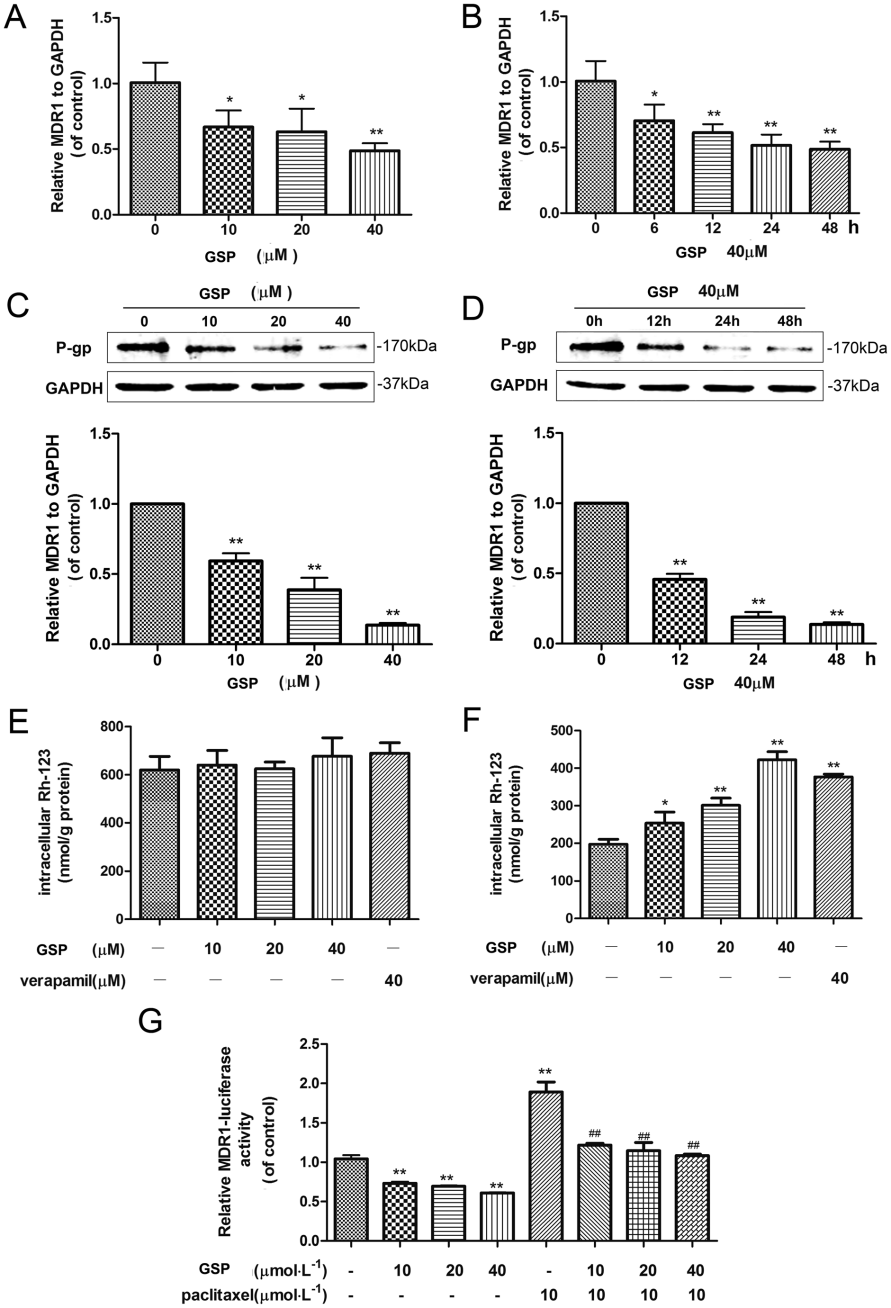
GSP inhibited the expression, function and transcription of MDR1 in A2780/T cells. (A and B) The effect of GSP interacted to MDR1 mRNA in A2780/T cells was determined by RT-PCR analysis. Cells were treated with different concentrations of GSP for 48 h (A) or different time-points for the concentration of 40 μM (B). Each column shows the mean ± SD of thee independent experiments, performed in triplicate. *Significantly different from untreated cells(P<0.05), **Significantly different from untreated cells (P<0.01). (C and D) The effect of GSP interacted to MDR1 protein in A2780/T cells was determined by Western blotting analysis. Cells were treated with different concentrations for 48 h (C) or different time-points for the concentration of 40 μM (D). The GAPDH band confirms the integrity and equal loading of protein. **Significantly different from untreated cells (P<0.01). (E and F) Effect of GSP on intracellular Rho-123 accumulation in A2780 (E) and A2780/T (F) cells. Cells were treated with 0∼40 μM GSP or 40 μM verapamil (positive control) for 24 h and then exposed to 10 μM Rho-123 for 90 min. intracellular Rho-123 accumulation was then measured. Each column shows the mean ± SD of thee independent experiments, performed in triplicate. *Significantly different from untreated cells (P<0.05), **Significantly different from untreated cells (P<0.01). (G) A2780/T cells were transiently transfected with plasmids harboring MDR1 reporter gene and treated with 0∼40 μM GSP with and without 10 μM paclitaxel for 24 h. The cells were lysed, and the MDR1 activities were measured by luciferase assay. Each column shows the mean ± SD of thee independent experiments, performed in triplicate. **Significantly different from untreated cells (P<0.01); ^##^Significantly different from paclitaxel-treated control cells (P<0.01).

Overexpression of MDR1 mRNA and protein levels is associated with the MDR phenotype. RT-PCR and Western blot analysis were performed to detect the change in MDR1 mRNA and protein levels upon treatment with GSP, respectively. As shown in Figure 3A and 3B, the MDR1 mRNA level markedly decreased in both dose- and time-dependent manner by GSP treatment. Similarly, GSP decreased the MDR1 protein level in both dose- and time-dependent manner (Fig. 3C and 3D). Thus, GSP suppressed P-gp/MDR1 expression in A2780/T cells.

### GSP significantly promoted the accumulation of Rho-123 in A2780/T cells

The P-gp-dependent efflux of fluorescent Rho-123 was extensively used to determine the efflux from drug-resistant cell lines expressing P-gp. The accumulation of Rho-123 in A2780/T cells was measured to determine whether the changes observed in MDR1 expression were correlated with the changes in P-gp function. Intracellular Rho-123 in A2780/T cells after treatment with GSP significantly accumulated in a dose-dependent manner (Fig. 3F), but the intracellular Rho-123 in A2780 cells insignificantly accumulated (Fig. 3E), reflecting the decreased P-gp efflux function in MDR1 overexpression cells rather than sensitive cells.

### GSP suppressed MDR1 expression by inhibiting NF-κB activity

To elucidate the effects of GSP on MDR1 promoter activity, A2780/T cells were transfected with MDR1 promoter reporter plasmids containing NF-κB responsive region and were then treated with various concentrations of GSP and paclitaxel. As a result, GSP was observed not only to have dramatically inhibited MDR1 promoter activity in a dose-dependent manner, but also to have dose-dependently suppressed paxlitaxel-activated MDR1 promoter activity (Fig. 3G).

**Figure 4:**
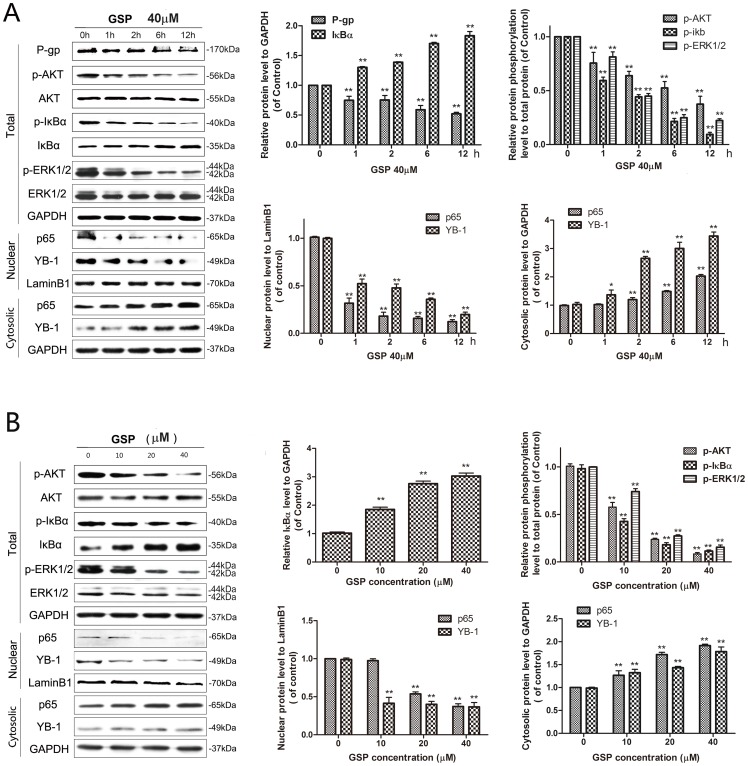
GSP down-regulated the expression of MDR1 by inhibiting NF-κB and MAPK/ERK medicated YB-1 activation. (A) A2780/T cells were incubated with 40 μM of GSP for indicated times. The lysates were subjected to Western blot analysis using anti-P-gp and the certain antibodies related to NF-κB and MAPK/ERK pathway. GSP inhibited P-gp, phospho-AKT, phosphor-ERK1/2 and phospho-iκBα levels with activated iκBα level. The nuclear and cytosolic protein was extracted from cell lysates. Western blot analysis was performed using the antibodies against nuclear p65, YB-1 and laminB1 as well as cytosolic p65, YB-1 and GAPDH. The GAPDH and phospho-protein relevant total protein band confirmed the integrity and equal loading of total protein and phospho-protein respectively. The Lamin B1 and GAPDH band confirms the integrity and equal loading of nuclear and cytosolic protein respectively. **Significantly different from untreated cells (P<0.01). (B) A2780/T cells were incubated with 0∼40 μM of GSP for 24 h. Phospho-AKT, phosphor-ERK1/2 and phospho-iκBα levels with activated iκBα level were performed by Western Blot. The nuclear and cytosolic protein was extracted from cell lysates. Western blot analysis was performed using the antibodies against nuclear p65, YB-1 and laminB1 as well as cytosolic p65, YB-1 and GAPDH. The Lamin B1 and GAPDH band confirms the integrity and equal loading of nuclear and cytosolic protein respectively. **Significantly different from untreated cells (P<0.01).

**Figure 5:**
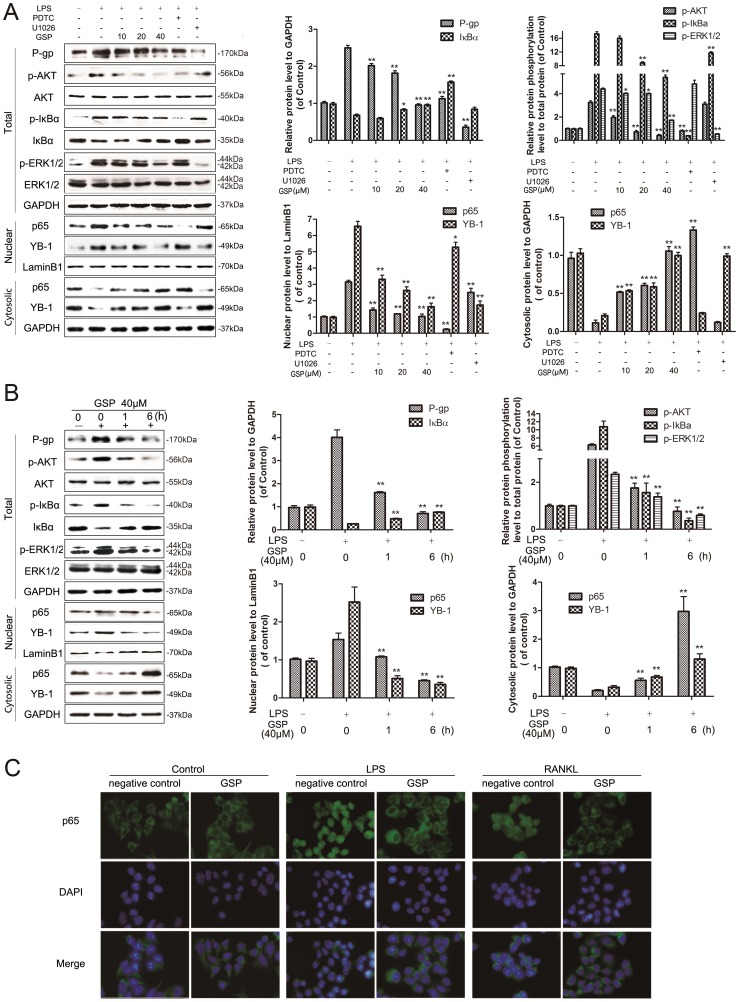
GSP attenuated LPS or RANKL induced over-expression of MDR1 by inhibiting NF-κB and MAPK/ERK medicated YB-1 activation. (A) A2780/T cells were incubated with 0∼40 μM of GSP for 6h then stimulated with or without 1 μg/ml LPS for 30min. 10μM PDTC and U1026 were used as NF-κB and MAPK/ERK inhibitor respectively. The lysates were subjected to Western blot analysis. GSP inhibited LPS-induced NF-κB, phospho-AKT, phosphor-ERK1/2 and phospho-iκBα levels with activated LPS-reduced iκBα level. The GAPDH and phospho-protein relevant total protein band confirmed the integrity and equal loading of total protein and phospho-protein respectively. *Significantly different from LPS-treated control cells (P<0.05); **Significantly different from LPS-treated control cells (P<0.01). (B) A2780/T cells were pre-incubated with 40 μM GSP for 0∼6h then simulated with or without 1 μg/ml LPS for 30 min. The nuclear and cytosolic protein were extracted from cell lysates. Western blot analysis were performed using the antibodies against nuclear p65, YB-1 and laminB1 as well as cytosolic p65, YB-1 and GAPDH. The Lamin B1 and GAPDH band confirms the integrity and equal loading of nuclear and cytosolic protein respectively. **Significantly different from untreated cells (P<0.01). (C) A2780/T cells treated with 1 μg/ml LPS or 50 ng/ml RANKL followed by pre-treatment with or without 40 μM GSP for 24 h were fixed for immunofluorescence test with staining with anti-p65 (green) and DAPI (blue). The pre-treatment with GSP before LPS or RANKL stimulation prevented the nuclear translocation of p65.

To evaluate whether NF-κB is involved in the suppression of MDR1 expression by GSP, the effect of GSP on NF-κB activity and LPS or RANKL induced NF-κB activation. Lysates of the GSP-treated cells were analysed by Western blotting with anti-phospho-ser32 of IκB-α, anti-phospho-ser473 of AKT and anti-p65 antibodies. GSP partially inhibited phospho-AKT and phospho-IκBα in a time-(Fig. 4A) and dose-(Fig. 4B)dependent manner. Consistent with this, the amount of the nuclear p65 subunit of NF-κB was significantly decreased with its increasing in nucleus (Fig. 4A and B). Furthermore, nuclear translocation of p65 induced by LPS and RANKL was evaluated by immunofluorescence. The LPS and RANKL treatment induced p65 nuclear translocation in A2780/T cells as shown in Figure 5C. However, the pre-treatment with GSP before LPS or RANKL stimulation prevented the nulear translocation of p65, indicating that GSP suppressed LPS and RANKL induced NF-κB activation. To further examine the GSP inhibitory of P-gp mediated by NF-κB, A2780/T cells were exposed to 40 μM GSP 0 to 6 h or 0∼40 μM GSP 24 h prior to incubation with LPS for 30 min. P-gp expression and activity were induced by LPS, and this effect was reduced in the presence of 40 μM GSP 6 h (Fig. 5A) or different concentrations of GSP 24 h(Fig. 5B). Also, GSP could inhibit LPS-induced phosphorylation of AKT and IκB-α as well as p65 nuclear translocation (Fig. 5A and B), further confirming GSP could inhibit the LPS-induced NF-κB activation in A2780/T cells. PDTC as a NF-κB activation inhibitor, showed the same tendency of GSP. These data indicate that GSP suppressed MDR1 by inhibiting NF-κB activation.

### GSP suppressed MDR1 involved inhibition of MAPK/ERK pathway mediated YB-1 nuclear translocation

The A2780/T cells treated with GSP demonstrated the time-dependent inhibition of ERK1/2 phosphorylation and YB-1 nuclear translocation (Fig. 4A), as well as, dose-dependent effect (Fig. 4B), indicating that GSP may suppress MAPK/ERK mediated YB-1 activation in A2780/T cells. Furthermore, to confirm the role of MAPK pathway in MDR1 suppression by GSP, the MAPK/ERK pathway were activated by LPS in A2780/T cells. Incubated with either different concentrations of GSP for 6 h or 40 μM GSP 0 to 6 h, A2780/T cells were treated with 1 μg/ml LPS for 30 min, the LPS treated A2780/T cells were significantly induced the MAPK/ERK pathway (Fig. 5A). Treatment with 40 μM GSP 0∼6 h (Fig. 5B) and 0∼40 μM GSP for 6 h (Fig. 5A) markedly inhibited the LPS-induced ERK1/2 phosphorylation and YB-1 nuclear translocation, compared with phosphor-ERK1/2 level and YB-1 nucleus expression levelin GSP-free group, respectively. ERK1/2 inhibitor U1026 was used as a positive control, showed the same tendency of GSP. These results indicate that GSP suppression of MDR1 by restraining the YB-1 activation via MAPK/ERK pathway inhibition.

## Discussion

P-glycoprotein, encoded by MDR1 and located on 7, q21.1 chromosome, has attracted great interest because of its crucial role in MDR in a variety of cancers [34], [35]. P-glycoprotein overexpresses in many cancer cells including ovarian cancer cells and is known to actively reduce the efficacy of chemotherapeutic agents and even result in treatment failure ultimately in ovarian cancer patients [36]. Therefore, blocking MDR1 is believed to enhance the efficacy of chemotherapy in these patients in previous studies. Different kinds of P-gp inhibitor developed from generation to generation. Although these agents worked successfully in vitro, most results of clinical trials were disappointing, because some of these synthetic compounds did not work in vivo or had unexpected side-effects. Due to the limited cytotoxicity and food-bored, experts turned their focus to natural plants sourced compounds.

Recently, a series of studies have reported the extensive bioactivities of GSP which used as a natural supplement for regulating oxidative balance. In these studies, GSP is found to reverse the MDR effect in blood-brain barrier by blocking the function of P-gp [30] but the MDR reversal effect of GSP in chemotherapeutic agent resistant cancer cells and even the mechanism of MDR reversal is not clear. Additionally, there's no report on the influence of P-gp expression by GSP, and also the mechanism is unknown. Because of the structural similarities of GSP with other dimmer flavonoids, which have been reported to reverse MDR by inhibiting both function and expression of P-gp [37], hence, it is reasonable to speculate that GSP may reverse MDR by blocking the expression of P-gp as well. Furthermore, Vayalil et al. have found that GSP inhibited epithelial-mesenchymal transition by NF-κB and MAPK pathway [38]. However, there's no report on whether NF-κB and MAPK/ERK pathway is involved in the inhibition of P-gp expression by GSP. Besides, co-expression of YB-1 and P-gp may be a promising relevant biomarker for unfavorable prognosis in ovarian cancer [39], the interventional effect of YB-1 on GSP regulating P-gp expression in ovarian cancer cells is also unknown yet.

Therefore, this study provides the first trial on GSP to explore its role in reversing MDR in paclitaxel resistant ovarian cancer cells by attenuating the expression and function of P-gp/MDR1. Our results showed that GSP markedly increased the cytotoxicity of paclitaxel in paclitaxel resistant P-gp overexpression A2780/T cells but faintly in paclitaxel sensitive A2780 cells, which indicates that GSP may reverses MDR by mediating overexpressed P-gp. Similarly, GSP also markedly increased the cytotoxicity of adriamycin in A2780/T but A2780, illustrating that GSP enhanced the cytotoxicity of paclitaxel is a non-specifical drug interaction but MDR reversal. Meanwhile, GSP significantly increases intracellular Rho-123 accumulation in A2780/T cells indicating that GSP inhibits the function of P-gp. Furthermore, we found that GSP down-regulated the expression of P-gp in both mRNA and protein level in a dose- and time-dependent manner, as we speculated. Additionally, GSP inhibited the MDR1 promoter activity and paclitaxel-induced MDR1 promoter activation. These results fully evidence that GSP has capable to suppress the activity of MDR1 transcription. The reduction of MDR1 expression probably at both translational and transcriptional levels has been proposed as one mechanism by which some agents reverse the MDR phenotype [40]. Thus, GSP reverses MDR phenotype by partly inhibiting both translation and transcription of MDR1, as a result of decreasing the function and expression of P-gp.

Inactive NF-κB dimmers, composed of p65 and p50 subunits, are sequestered in the cytoplasma in association with the inhibitory molecules of the IκB family. Stimulation of cells with LPS and RANKL causes phosphorylation of the inhibitor IκBα, leading to its polyubiquitination and proteasome-mediated degradation and the release of active NF-κB [41]. Furthermore, AKT phosphorylation mediates the phosphorylation of IκBα to activate NF-κB pathway [42]. NF-κB induces the transcription of MDR1 by translocating into nucleus binding the respondent region of MDR1 promoter and then influences the expression of P-gp [20], [43]. The current study was designed to assess the possible role of NF-κB activation in GSP reducing the expression of P-gp. First, GSP was shown to inhibit NF-κB pathway by restraining the AKT and IκBα phosphorylation so as to control decomposition of IκBα, the inhibitor of NF-κB. Then, GSP was found to increase the expression of p65 (a NF-κB subunit) in cytosol while decrease nuclear p65 expression to block the nuclear NF-κB mediated MDR1 transcription. Moreover, GSP obstructed LPS and RANKL induced NF-κB nuclear translocation, manifesting its subunits reversely translocated from nucleus to cytoplasma. These findings indicate that the inhibition of NF-κB activation may be involved in GSP suppressing the expression of P-gp.

On the other hand, YB-1 is an oncogenic transcription/translation factor that is over-expressed in a number of cancer types. Previous studies have shown that YB-1 activated by Akt and the activation of MAPK/ERK appeared YB-1 of normally localizing in the cytoplasm translocated into nucleus, leading to activation of the MDR1 expression [44]. Additionally, many reports supported that MDR1 gene could be activated by external signals via MAPK/ERK [16]. In this study, GSP reduced the phosphorylation of ERK1/2 and inhibited the YB-1 nuclear translocation while GSP suppressed P-gp expression. Furthermore, other studies showed that the specific inhibitors of ERK significantly reversed P-gp-mediated MDR [11], [16], [17]. Thus, GSP is prospected to down-regulate the expression of P-gp probably associated with the inhibition of MAPK/ERK pathway. To test this prospection, LPS was also used in our study to induce P-gp, MAPK/ERK and YB-1 activation so as to identify whether GSP can attenuate the induced phosphorylation of ERK as an ERK inhibitor U1026. Our findings showed that GSP attenuated LPS-induced ERK1/2 phosphorylation and YB-1 nuclear translocation respectively, which further testified our hypothesis, suggested that GSP suppressed P-gp expression was regulated by YB-1, in part, through regulating the action of MAPK/ERK.

Taking together, our current experiments show that GSP significantly increases the efficacy of paclitaxel and adriamycin in A2780/T cells by reversing the phenotype of MDR via blocking the function of P-gp and inhibiting the expression and transcription of MDR1. Furthermore, GSP can suppress the NF-κB activity and MAPK/ERK pathway mediated YB-1 nuclear translocation, which may have a correlation with the expression-down-regulated P-gp. Although we have preliminarily verified the possible mechanism of GSP inhibiting P-gp, the specific target of GSP interacting P-gp via NF-κB and MAPK/ERK pathway is unknown. Moreover, our results are only limited to human ovarian cancer cells of A2780/T and A2780 in vitro. It's worth our further investigating whether GSP could reverse MDR in other ovarian cancer cells or another cancer cell model as well as the in vivo assay.

In conclusion, our data demonstrates that GSP reverses MDR in A2780/T cells by inhibiting both function and expression of P-gp and inhibits P-gp expression in A2780/T cells possibly via the suppression of NF-κB activity and MAPK/ERK pathway medicated YB-1 activation. This study first verifies the effect and mechanism of a natural supplement GSP reversing MDR by interacting P-gp, suggesting that GSP may be used in combination with conventional P-gp substrate chemotherapeutic drugs to overcome MDR in ovarian cancer patients, providing a new use of such natural supplements as a chemo-sensitizer.
